# A Guided Vehicle under Fire Conditions Based on a Modified Ultrasonic Obstacle Avoidance Technology

**DOI:** 10.3390/s18124366

**Published:** 2018-12-10

**Authors:** Sen Li, Chunyong Feng, Xiaoge Liang, Hengjie Qin, Haihang Li, Long Shi

**Affiliations:** 1 School of Building Environment Engineering, Zhengzhou University of Light Industry, 5 Dongfeng Road, Zhengzhou 450002, Henan, China; lisen@zzuli.edu.cn (S.L.); fengchunyong@zzuli.edu.cn (C.F.); liangxiaoge@zzuli.edu.cn (X.L.); qhj102432@zzuli.edu.cn (H.Q.); 2 School of Quality and Safety Engineering, China Jiliang University, 258 Xueyuan Road, Hangzhou 310018, Zhejiang, China; lihaihang@cjlu.edu.cn; 3 Civil and Infrastructure Engineering Discipline, School of Engineering, RMIT University, Melbourne, VIC 3000, Australia

**Keywords:** Ultrasonic obstacle avoidance technology, guided vehicle, fire condition, baffle calibration method

## Abstract

Low visibility and hot smoke environment under fire conditions can largely hamper the related fire rescue processes. Ultrasound obstacle avoidance technology is then useful for guidance. However, the biggest challenge of adopting ultrasound technology comes from accurate distance measurements under the disturbances of high temperature and soot particle concentration. It is critical to measure the propagation speed under the complicated fire conditions. Therefore, in this study, a baffle calibration method was proposed to improve the accuracy of distance measurement of an obstacle. The method is based on two ultrasound measurement systems, while one is used to calibrate the propagation speed of ultrasound based on the fixed distanced baffle and the other is for the dynamic measurement of obstacle distance based on the calibrated speed. The viability of this method on the guided vehicle was confirmed based on the experiments. From its comparison to those existing methods, such as constant speed and temperature compensation methods, it was known from that the proposed baffle calibration method provides the best prediction. It was obtained that the maximum errors based on the baffle calibration method are 2.75% and 2.62% under the two representative fire scenarios, respectively, which are much lower than those of constant speed (7.81% and 8.4%) and temperature compensation methods (10.4% and 5.12%).

## 1. Introduction

**Figure 1:**
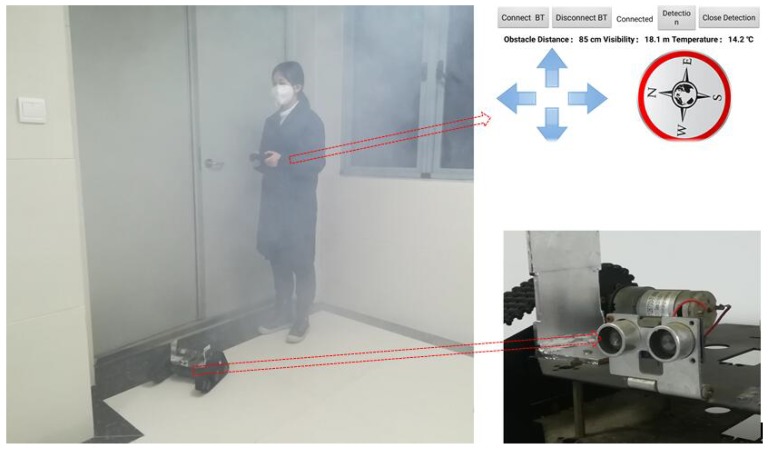
Guided vehicle guides the walking under fire conditions (**left**), which includes the distance measurement module (**bottom right**) and remote-control system (**upper right**).

Due to the complicated fire situations, occupants may be trapped in a fire that the related fire rescue processes are then critical to save lives. During the processes, it is very important for those firefighters to enter the building timely and correctly by avoiding the obstacles. However, those fire rescue activities are difficult due to high temperature and low visibility environment under a large amount of smoke. The visibility could decrease sharply when the density of smoke exceeds a certain level. For example, people could only keep their eyes open for a short time and tears run so heavily in thick smoke under 0.5 m [1]. Due to the limited time for fire rescue under the rapid fire spread inside buildings, the key to successful fire rescue is to avoid the obstacle when the visibility is often near zero under fire conditions [2,3]. Currently, a popularly adopted way is often based on a guided vehicle, as shown in Figure 1. The guided vehicle is positioned in front of those firefighters, while those monitoring modules in it then detect the obstacle and provide guidance for them based on the information on the monitor of the remote control.

The distance measurement of the obstacle is the determining factor on the performance of the guided vehicle. Recently, obstacle detection has attracted increasing attention from researchers in order to accurately predict the obstacle distance under fire conditions. For example, Kim et al. [4] and Starr and Lattimer [5] developed a multi-spectral vision system by using sensor fusion between stereo thermal infrared (IR) vision and frequency modulated-continuous wave (FMCW) radar. Kiss and Szirányi [6] and Li [7] used a proper orthogonal decomposition to filter smoke occlusions in fire image streams and the effectiveness of the technique has been demonstrated by experiment. Sales et al. [8] utilized a laser rangefinder and sonar sensors combined with a vision system and developed a navigation robot system. Starr and Lattimer [9] performed an experimental study to quantify the performance of eleven common robotic navigation range finding technologies and camera systems in a fire smoke environment. The results are shown in Table 1. It was indicated that instruments operating at higher wavelengths can outperform those operating at lower wavelengths.

For those technologies with high wavelength, ultrasound avoidance technology shows its advantages for the related measurement under fire smoke environment. For example, those infrared vision systems are very affected by the surrounding high-temperature objects, failing to detect the obstacles under the limited resolution. Radar technology has been popularly adopted in military areas that the related instruments are usually heavy and with high cost, which is currently not viable for fire rescue. When comparing to the infrared vision and radar systems, ultrasound technology has been largely adopted in many critical areas such as oceanology, medical treatment, and geography. The ultrasound technology is quite mature for civil industries, while it can be implemented in a small device with much lower cost. Therefore, based on these characteristics, ultrasound technology is highly viable to solve the current issue for obstacle detection in a fire smoke environment.

The ultrasound as a mechanical wave can be largely affected by the media of the environment, such as hot smoke. While the studies on the propagation speed of ultrasound in different media are quite mature, the situation for fire smoke environment is quite complicated and challenging as it includes many influencing factors, such as hot smoke, soot, and floating matters [10]. Therefore, it is still a challenge for an accurate obstacle distance measurement based on ultrasound in complicated and violent fire conditions.

Therefore, in this study, a baffle calibration method was proposed to correct the propagation speed of ultrasound for the accurate prediction of obstacle distance under fire conditions. In the following contents, Section 2 addressed the typical influencing factors on the measurement of obstacle distance, Section 3 introduced the principles of baffle calibration method, Section 4 focused on the experimental methodology and the relevant experimental results, and Section 5 provided a further discussion and analysis on the experimental results, and the last section is the concluding remarks of this study.

## 2. Influencing Factors on Obstacle Distance Measurement

The typical methods for ultrasonic distance measurement include time-of-flight (TOF) technique, phase detection method, and amplitude detection method. Among these methods, the phase detection method could provide the highest prediction accuracy, but it is limited to a short distance. The media is also found showing obvious influences on the implementation of the amplitude detection method, while the prediction accuracy is low at the movement. The TOF technique can provide good prediction accuracy with an acceptable level of distance measurement. The TOF technique can be implemented by a simple electro circuit, which is the reason for its wide adoption in the industries [11]. In this study, the TOF technique is also adopted for the related experimental measurement.

The distance measurement of the TOF technique is based on Reference [12],

(1) S=ct/2

where *S* is the distance between the measurement spot and the obstacle; *c* is the speed of sound; and *t* is the return time between the emitter and obstacle.

It can be known from Equation (1) that the speed of ultrasound is the key for accurate measurement. As a mechanical wave, the propagation speed of ultrasound could not keep constant all the time, which is very dependent on the media. To provide an accurate measurement, it is critical to address the relationship between the propagation speed and surrounding environment.

### 2.1. Relationship between Propagation Speed and Temperature

The propagation speed of ultrasound reflects, to some extent, the compression characteristics under the disturbance from the media. The propagation speed can be obtained by:

(2) $$c=\sqrt{\frac{dP}{d\rho }}$$

where *P* is the pressure of the media; and *ρ* is the density of the media.

For short-wave ultrasound in the ideal gas, its propagation speed can be expressed based on the assumption that the whole process is adiabatic:

(3) $$c^{2}=\gamma \frac{P}{\rho }$$

where *γ* is the ratio between the specific heat capacity of the air at constant pressure and constant volume.

After combining the ideal gas’s law, Equation (3) can be given by Reference [13]:

(4) $$\mathrm{PV}=\frac{M}{\mu }\mathrm{RT}$$

(5) $$c=\sqrt{\gamma \frac{R}{\mu }T}$$

where *μ* is the molar mass of the air; and *R* is the gas constant.

There are still lots of unknowns in Equation (5) that will encounter difficulties in the practical implementations. It can be known from this equation that among those influencing factors (e.g., temperature, relative humidity, and gas species), temperature shows the biggest influence on the propagation speed. The propagation speed at 0 °C has been largely adopted as a benchmark. The calibration model of the speed can be given by Reference [14]:

(6) $$c\approx 331.4+0.607T_{c}$$

where *T_c_* is the temperature of the media, °C.

Based on the calibration model, the usually adopted way is to install a temperature transducer for the measurement of temperature to calibrate the measurement of obstacle distance.

### 2.2. Relationship between Propagation Speed and Soot Concentration

While the calibration model based on temperature can be widely adopted in the relevant devices, it assumes the media as an ideal gas, while temperature is solely considered in the model. While the related error based on the assumption can be usually ignored, the prediction under some special conditions such as rain and haze still encounter non-negligible error based on this calibration model [15]. This is mainly because of the influences from liquid and solid particles under these complicated conditions. The fire smoke environment not only include those hot smoke, but also many soot particles. The ultrasound distance measurement can only be adopted for fire smoke environment unless the influences of soot particles on its propagation speed are clearly known. However, this is not the case currently.

The studies on addressing the influences have been largely focusing on the measurement of emulsion concentration [16,17,18]. Currently, the Epstein-Carharts-Allegra-Hawley’s model (ECAH) [18] was usually adopted to address the influences of particles on the propagation speed of ultrasound. ECAH mainly considers the attenuation of ultrasound due to the heat dissipation, viscosity, diffuse scattering, and inner absorption. McClements [19] obtained the propagation speed of ultrasound in a media with particles by assuming that the wavelength is much bigger than the particle size,

(7) $${\left(\frac{k_{s}}{k}\right)}^{2}=1+\frac{3\varphi }{jk^{3}R^{3}}\overset{\infty }{\underset{n=0}{\sum }}\left(2n+1\right)A_{n}$$

(8) $$k_{s}=\omega /c_{s}+j\alpha_{s}$$

(9) k=ω/c+jα

(10) $$A_{0}=j{\left(kR\right)}^{3}\left(\rho k_{s}{}^{2}/\rho_{s}k^{2}-1\right)/3-k^{2}RcT\rho \tau H{\left(\beta /\rho C_{p}-\beta_{s}/\rho_{s}C_{ps}\right)}^{2}$$

(11) $$A_{1}=\frac{-j{\left(kR\right)}^{3}}{3}\frac{\rho -\rho_{s}}{3\rho +2\left(\rho_{s}-\rho \right)/\left[1+3\left(1+j\right)\delta_{v}/2R+3j\delta_{v}{}^{2}/2R^{2}\right]}$$

(12) $$H={\left\{\frac{1}{1-jz}-\frac{\tau }{\tau_{s}}\times \frac{tanz_{s}}{tanz_{s}-z_{s}}\right\}}^{-1}$$

(13) $$z=\left(1+j\right)R/\delta_{t}$$

(14) $$\delta_{t}=\sqrt{\frac{2\tau }{\omega \rho C_{p}}}$$

(15) $$\delta_{\nu }=\sqrt{\frac{2\mu }{\omega \rho }}$$

where *n* is 2, namely only considering the thermal dissipation and viscosity; *k* is the wavenumber of the media; *φ* is the volume fraction of the particles; *α* is the attenuation coefficient; *c* is the propagation speed of ultrasound; *R* is the radius of the particles; *ω* is the angular frequency; *ρ* is the density; *T* is the absolute temperature; *τ* is the thermal conductivity; *β* is the thermal expansion coefficient; *C_p_* is the specific heat capacity at constant pressure; and *μ* is the shear viscosity. For those parameters with subscript s are for the particle, while no subscript means the media.

In Equations (7)–(15), only *α_s_* and *c_s_* are unknowns, while the other 14 parameters are all given or confirmed for the calculation. The large number of parameters complicates the relevant calculation, which largely affects the related implementations in the industries.

Those soot particles in fire smoke environment usually do not belong to a single type of matter, which is a combination of products from both complete and incomplete combustion and also water vapor. Considering the complicated components of the media, a theoretical model to accurately predict the propagation speed of ultrasound becomes quite difficult. It is also known that the propagation speed is also affected by the concentration and size of these particles, which are also changing with time. Therefore, at the moment, it is quite difficult to predict the propagation speed of ultrasound in fire smoke environment based on the existing theoretical models. Therefore, a baffle calibrate method was proposed in this study, where the details can be seen in the following contents.

## 3. Baffle Calibration Method

**Figure 2:**
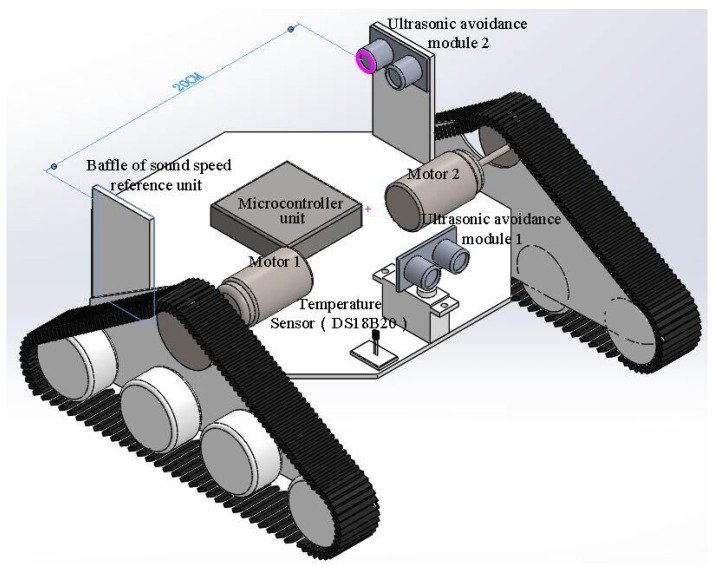
Guided vehicle based on the proposed baffle calibration method.

The baffle calibration method was proposed and adopted in the guided vehicle based on the thought of “reference speed”. The guided vehicle based on the baffle calibration method can be seen in Figure 2. The whole system includes vehicle, microcontroller unit, and ultrasound avoidance module. The microcontroller unit includes a microcontroller, a motor driving module, Bluetooth communication module and so on. The microcontroller used the STC15W4K16S4, which was supplied by Shenzhen Hongjing Technology, with a machine cycle of 1T, four isolated high-speed asynchronous series communication port, seven timers, and five 16-bit reloadable timer/counter. It supports a voltage within 2.5–5.5 V, with an operation environment of Keil µVision4. The motor driving module used L298N as the main chip, with a driving voltage of 5–35 V, which was adopted by the motors 1 and 2, as shown in Figure 2. It shows the advantage of high power, low heat generation, and high reliability. The Bluetooth communication module adopted the HC-05 from Guangzhou Huicheng Technology for the wireless data transportation, with a working frequency of 2.4 GHz and modulation of GFSK. The maximum transmission power is 4 dBm with a receiving sensitivity of −85 dBm. It was also equipped with a PCB antenna for communication within 10 m.

**Figure 3:**
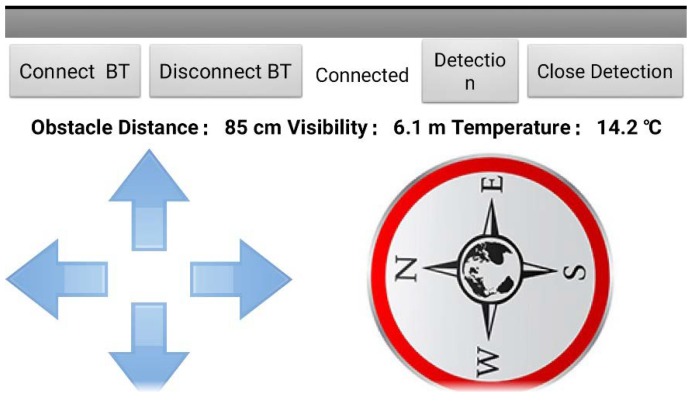
The interface of the remote control system.

The controller of the guided vehicle is based on Android, using the Bluetooth for data transportation. The interface of the controller can be seen in Figure 3. Through the controller, the guided vehicle can be moved, showing the related information, such as smoke temperature, visibility, and distance of the obstacle. The firefighters can be guided based on this information.

It can be seen that the guided vehicle shown in Figure 2 contains two ultrasound avoidance modules, where module 1 is for the measurement of obstacle distance and module 2 with the baffle at the front is to determine the reference speed under various scenarios. The distance between module 2 and the baffle was fixed at 20 cm. The reference speed can be obtained by the return time and the distance, which is given by:

(16) c=2S/t

where the *S* equals to 20 cm. As both modules are very closely located in the environment, it is then assumed that the propagation speed are the same. The reference speed based on module 2 can be then used for the measurement of the obstacle distance of module 1.

**Figure 4:**
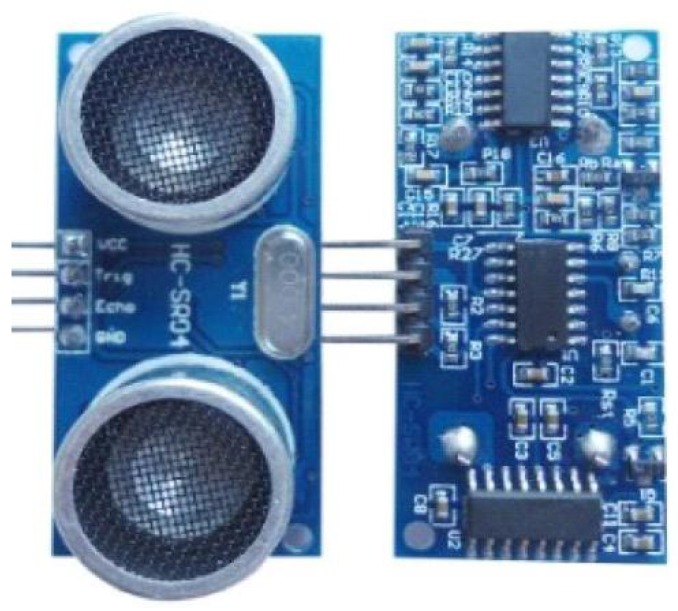
Ultrasound module of HC-SR04: Front (**left**) and back (**right**) faces.

The two modules in the system are the same type (HC-SR04) supplied by Shenzhen Jieshen Technology Co., Ltd. (Shenzhen, China), in order to avoid the errors raised by the electro circuit. The module can be seen in Figure 4, while the main technical parameters obtained from the supplier are listed in Table 2.

**Figure 5:**
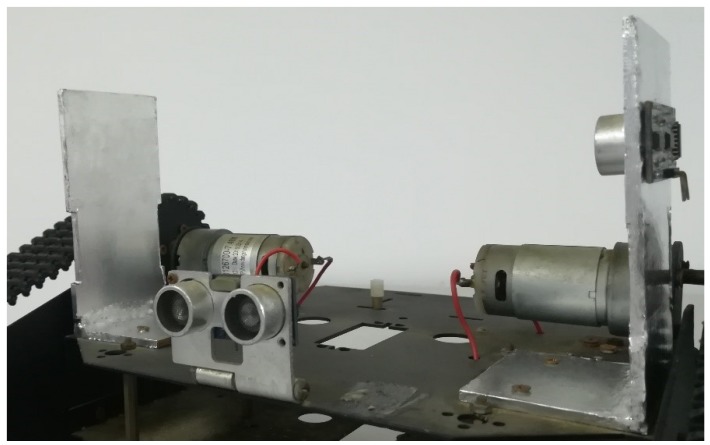
The Photograph of the proposed guided vehicle.

To benefit the reflection of the ultrasound, the baffle in front of the module 2 should be those materials with smooth surface and hardness. Furthermore, the material should consider the high-temperature environment that the melting point should be high. Considering the above, aluminum plate was selected for this study, as seen in Figure 5. The advantage of the baffle calibration method is that there is no need to measure directly the temperature and soot concentration in the changeable fire smoke environment, while it only needs to measure the transportation time between the two modules. It is not only can improve the prediction accuracy, but also fills the gap under the temporary absence of high-accurate theoretical models for fire conditions.

## 4. Validation by Experiments

### 4.1. Experimental Methodology

**Figure 6:**
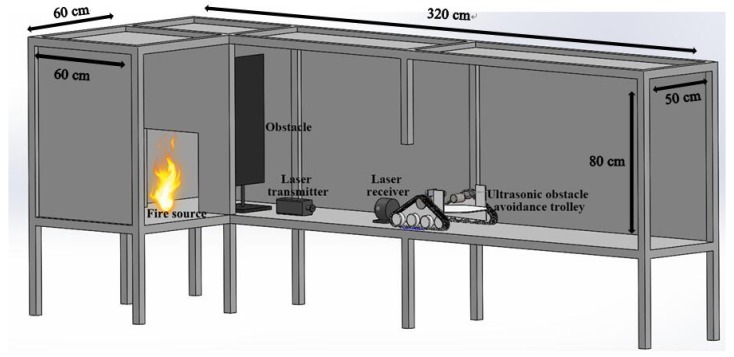
Schematic of reduced-scale test room model.

To validate the baffle calibration method, a test platform was developed based on a reduced-scale test room model, as shown in Figure 6. The test room model includes a room (area of 60 cm × 60 cm) and a corridor (320 cm × 50 cm) with a ceiling height of 80 cm, while the fire source was located in the room. After the ignition of the fire source, the smoke spread from the room to the corridor through the door between them. The corridor was used to simulate a fire smoke environment with hot smoke and soot particles. The proposed guided vehicle was put inside the corridor.

**Figure 7:**
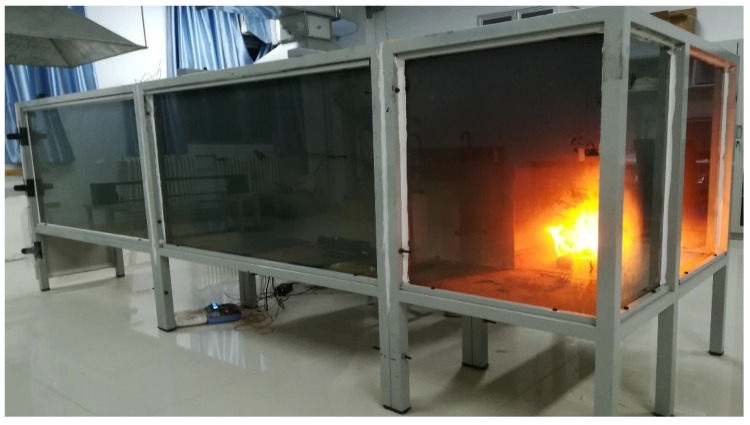
The photograph of the fire test room model.

The whole test room model was sealed except the air supply opening at the bottom of the room. After the ignition, the air supply opening provides enough air to support the stable burning process. The air supply opening was shut down when the fire is extinguished. The test room model was built based on a steel frame and Magnesium fireproof board for the wall. To help the observation, one side of the model adopts fireproof glass, while the photograph can be seen in Figure 7.

**Figure 8:**
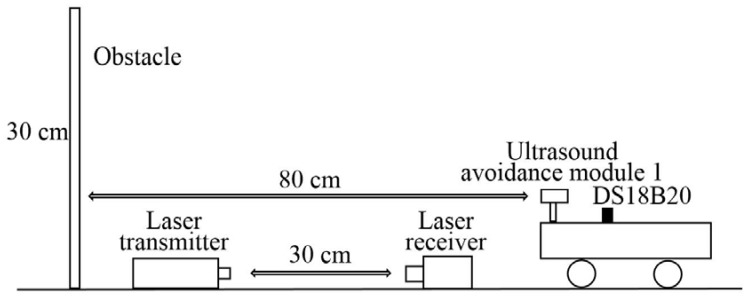
The layout of experimental equipment in the corridor.

The guided vehicle was positioned inside the corridor, while a timber board with a smooth surface was placed in front of the device, considered as an obstacle. The timber board is with a dimension of 20 cm (W) × 30 cm (L) × 2 cm (D). The distance between the guided vehicle and the timber board was fixed at 80 cm, as shown in Figure 8. The measured distances were compared with the fixed distance (i.e., 80 cm) in the following contents.

In order to determine the influences of temperature and soot concentration on the ultrasound propagation speed, the room test model was also equipped with laser and temperature sensor for the related measurement. A temperature sensor (DS18B20) was adopted in the guided vehicle (Figure 8) for the temperature measurement with a range of −55 °C~+125 °C. The soot concentration was measured by a group of laser transmitter (HNLS008R) and receiver (PDA36A), while the principle is to reflect the soot concentration based on the attenuation of the laser intensity. The distance between the laser transmitter and receiver was fixed at 30 cm, as seen in Figure 8. The attenuation of the laser intensity can be considered as the extinction coefficient of the fire environment,

(17) $$E=\frac{1}{L}\ln(\frac{U_{0}}{U})$$

where *E* is the extinction coefficient, 1/m; *L* is the distance between the laser transmitter and receiver, which is 0.3 m in this study; *U*_0_ is the output voltage under ambient condition, *V*; and *U* represents the output voltage under the fire smoke environment, *V*.

In the fire safety area, the visibility has also been frequently adopted to reflect the soot concentration, where the relationship between the visibility and extinction coefficient can be given by Reference [20],

(18) $$V=\frac{C}{E}$$

where *V* is the visibility, m; and *C* is the constant, while *C* is within 2~4 for those non-luminous objects. In this study, *C* is considered as 2.3 [20].

### 4.2. Distance Measurement

**Figure 9:**
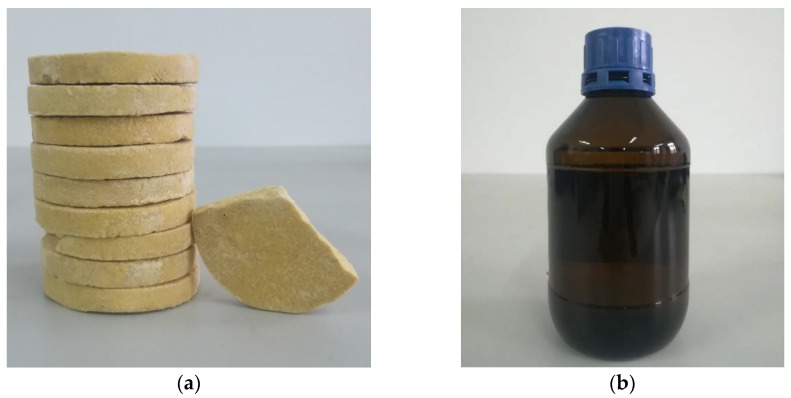
The two materials to simulate fire smoke: (**a**) Smoke cake with Ammonium and (**b**) n-heptane.

The smoke under fire conditions can be normally divided into two types: one is the smoke with high concentration but low temperature, which is usually at the early stage of spontaneous ignition; and the other is the smoke with low concentration but high temperature, which can be seen during the fire development stage. Therefore, based on this, the first type of smoke with high concentration and the low temperature was simulated by smoke cake with Ammonium Chloride (Figure 9a). To simulate the smoke with a low concentration and high temperature, n-heptane was burning as the fire source, as shown in Figure 9b.

Furthermore, the measurements based on the temperature compensation and constant speed methods were performed to enable the comparison with the proposed baffle calibration method. The temperature compensation method was based on the temperature sensor in the guided vehicle, where the related calculation was based on Equation (6). For the constant speed, the propagation speed of the ultrasound was assumed to be kept at 340 m/s, ignoring the influences from the changed temperature and soot concentration.

#### 4.2.1. Fire Smoke with High Concentration and Low Temperature

**Figure 10:**
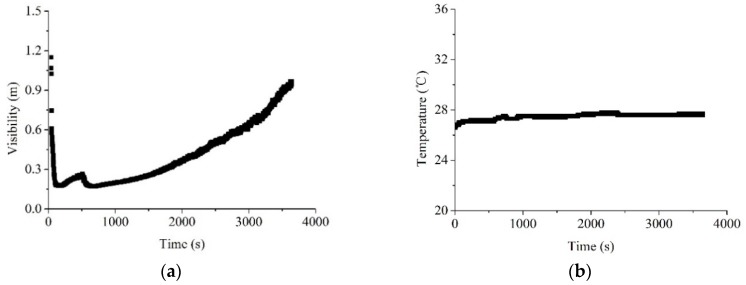
Visibility (**a**) and temperature (**b**) histories for the smoke produced by smoke cake.

Under the smoke produced by the smoke cake, the visibility and temperature along the time can be seen in Figure 10. During the first 200 s, a large amount of smoke was produced, resulting in a rapidly decreasing visibility. After 200 s, the visibility keeps increasing due to the consumption of the smoke cake. This is the stage with stable data and uniform distribution of smoke inside the corridor. Thus, the data analysis was mainly on this stage. It can be seen that the smoke produced by smoke cake show limited influence on the temperature, where only a 1 °C temperature rise was observed. This is quite consistent with the early stage of fire development, which also serves as an excellent media to determine the influences of smoke concentration on the propagation speed of ultrasound. It should be mentioned that the maximum visibility during the whole experimental period is about 1.2 m, as seen in Figure 10, which provides a very good condition to simulate fire smoke environment.

**Figure 11:**
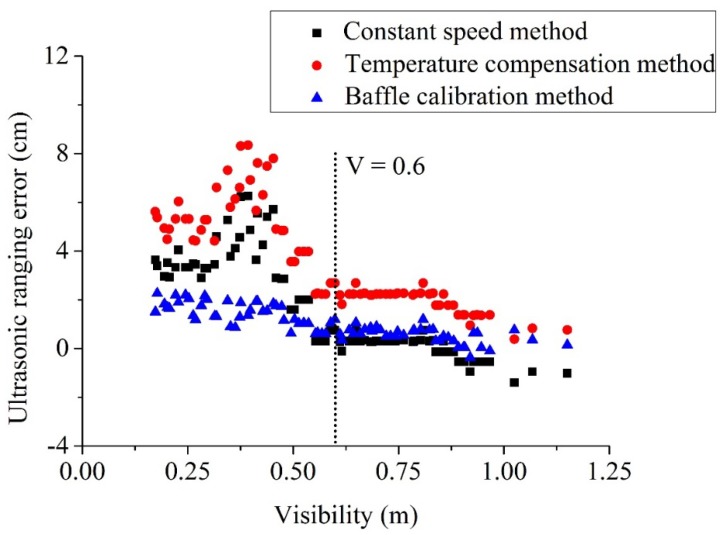
Measurement errors of three typical methods along the visibility, the measurement errors can be divided into two stages at a visibility of 0.6 m.

Figure 11 shows a comparison of measurement errors of three methods, including the proposed baffle calibration method, and the existing temperature compensation and constant speed methods. It can be seen from this figure that the measurement errors along the visibility can be further divided into two stages, with a turning point at a visibility of 0.6 m. When the visibility is larger than 0.6 m, the measurement errors for all the three methods are keeping at a relatively low level. The measurement errors for baffle calibration and constant speed are approaching zero, while for the temperature compensation, the errors are relatively high (around 2 cm). When the visibility is lower than 0.6 m, it can be seen the measurement errors for both temperature compensation and constant speed keep increasing, while the errors based on baffle calibration keeps at a relatively lower level all the time. Based on this, it can be seen that the measurement based on baffle calibration is quite stable and reliable under various scenarios.

The statistical data listed in Table 3 also prove the statement that the baffle calibration method shows its advantages when comparing to the other two methods in terms of stability and accuracy. Those errors listed in Table 3 are the absolute errors (always positive), while the mean absolute deviation is the average values of all the absolute errors. The related calculation can refer to Equation (19). The mean absolute deviation is a better indicator to reflect the real errors when compared to the average errors as there is no summation of negative and positive errors during the calculation. The calculation of standard deviation is based on Equation (20). The maximum relative error represents the ratio between the maximum error and the distance (e.g., 80 cm).

It can be seen from Table 3 that: (a) The battle calibration method produces minimal errors based on the maximum and minimal error and means absolute deviation. Based on the mean absolute deviation (MAD), it shows a sequencing of temperature compensation (3.6 cm) > constant speed (1.9 cm) > baffle calibration (1.0 cm); (b) a sequencing based on standard deviation can be obtained as temperature compensation (2.0 cm) > constant speed (1.8 cm) > baffle calibration (0.6 cm); and (c) according to the maximum relatively error, it is known that temperature compensation (10.4%) > constant speed (7.81%) > baffle calibration (2.75%). According to the above analysis, it is known that the proposed baffle calibration method provides the best prediction, followed by constant speed and temperature compensation methods, respectively, in a fire smoke environment.

(19) $$MAD=\frac{1}{N}\sum_{i=1}^{N}\left|S_{i}-80\right|$$

(20) $$\sigma =\sqrt{\frac{1}{N}\overset{N}{\underset{i=1}{\sum }}{\left(S_{i}-MAD\right)}^{2}}$$

*S_i_* in Equations (19) and (20) is the distance measurement for each ultrasonic ranging, and *N* is the time of measurement, and 80 is the actual distance value between the ultrasonic transducer and the obstacle.

#### 4.2.2. Fire Smoke with a Low Concentration and High Temperature

**Figure 12:**
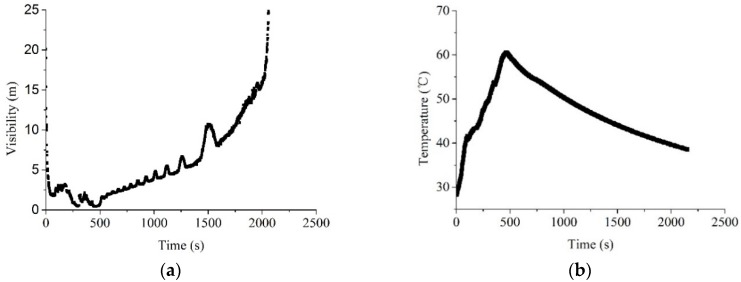
Visibility (**a**) and temperature (**b**) histories for the smoke produced by n-heptane.

The visibility and temperature histories for those smoke produced by n-heptane can be seen in Figure 12. The first 400 s is the period for the burning of n-heptane, where the visibility rises rapidly with a significant increase in the temperature. The fire smoke was spreading from the room to the corridor, which is quite unstable. After 400 s, the burning of the n-heptane stops when it was consumed. It can be seen that the visibility rises slowly with a smoothly decreased temperature. This is the period adopted for the measurement of the distance, where the maximum temperature can be as higher as 60 °C.

**Figure 13:**
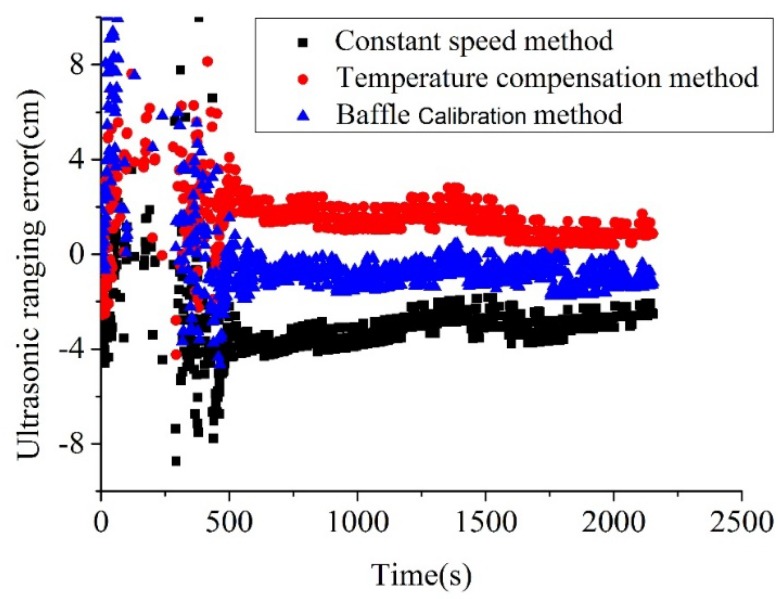
Measurement errors of the three methods along the time.

Under the smoke produced by n-heptane, the propagation speed of the ultrasound is dependent on both smoke concentration and temperature. At the moment, the existing models in the literature are difficult to accurately address the influences from the individual factor. This is the reason why the measurement error along the time was provided, as shown in Figure 13. It can be seen that the measurement errors based on all the three methods are quite unstable during the first 400 s, which is the unstable period for rapid spreading of the smoke. After 400 s, it was observed that the measurement errors are quite stable, where the errors keep decreasing along the time. Based on the comparisons of these three methods, it can be seen that the baffle calibration method shows the advantage, with near-zero errors during the stable period. The measurement errors for temperature compensation methods are positive, but they are negative for constant speed methods.

The comparison of the three methods can be seen in Table 4 based on the statistical analysis. It can be seen that the proposed baffle calibration method produces minimal measure errors in terms of all the three parameters, including the maximum and minimal error and mean absolute deviation. Differently, under the situation, the temperature compensation method is showing a relatively better prediction than those of the constant speed method.

### 4.3. Results and Discussion

Based on the experiments of the two scenarios, it can be known that the baffle calibration method shows its advantages in terms of stability and accuracy when comparing to the temperature compensation and constant speed method. Several phenomena happened during the experiments are further addressed here.

According to the above analysis, it is known that the measurement accuracy of ultrasound is much dependent on its propagation speed. The relationship between the propagation speed and temperature can be seen in Equation (6), which indicates that the propagation speed increases under a high temperature. The influences of the soot particles on the propagation speed are quite complicated. Challis et al. [21] investigated the particle environment such as polystyrene, silica, and TiO_2_. It was obtained that the propagation speed is bigger than that in water, while the speed under TiO_2_ is relatively smaller. It was observed from Figure 11 that the measurement errors based on constant speed method are positive, reflecting a relatively bigger propagation speed for ultrasound. It is indicated that real propagation speed under fire smoke environment should be lower than the assumed constant speed, namely 340 m/s. Therefore, it can be concluded that fire smoke could decrease the propagation speed of the ultrasound.

This conclusion is quite useful to explain the experimental phenomenon. For example, under smoke with high concentration and low temperature, the biggest errors can be found from the temperature compensation method. This is mainly because this method only considers the temperature variation, so the predictions are then higher when it shows a relatively higher temperature than the ambient environment. Under a smoke environment with a low concentration and high temperature, the measurement errors of the temperature compensation and constant speed are positive and negative, respectively. It is then indicated that the positive measurement errors are due to the ignorance of the soot particles. It should be mentioned that the temperature compensation method provides relatively high prediction accuracy for the high-temperature environment, which indicates the temperature plays an important role in affecting the propagation speed of ultrasound.

Therefore, due to the complicated conditions under fire smoke environment, it is still a challenge to theoretically predict the propagation speed of ultrasound under various smoke concentration and temperature. This gap can be filled by the proposed baffle calibration method in this study, which shows advantages in providing reliable and accurate predictions. The method is helpful in assisting the fire rescue.

## 5. Conclusions

While many techniques have been adopted in the industries about obstacle detection, the related studies under fire smoke environment are relatively limited to benefit effective rescue under fire smoke environment. In this study, a guided vehicle based on baffle calibration method was proposed and validated by experiments with two fire scenarios, including early stage (smoke with high concentration and low temperature) and development stage (smoke with a low concentration and high temperature). Based on its comparisons with temperature compensation and constant speed methods, it is known that the proposed baffle calibration method shows its advantages in terms of both reliability and accuracy. It is obtained that the maximum relative errors of the proposed baffle calibration method are 2.75% and 2.62% under the two fire scenarios, which are much lower than constant speed (7.81% and 8.4%) and temperature compensation methods (10.4% and 5.12%). The proposed baffle calibration method provides a practically useful tool for fire rescue.

## Figures and Tables

**Table 1:** Applicability of detection devices under fire smoke environment [9].

Detection Device	Operating Wavelength	Applicability
Regular camera	~0.4–0.7 μm	Error when visibility < 8 m; and Failure when visibility < 1 m
Infrared night vision device	~0.4–0.8 μm	Failure when visibility < 4 m
Thermal infrared camera	8–14 μm	Limited influence
Xbox KinectTM Depth Sensor	~0.830 μm	Error when visibility < 8 m
Light Detection and Ranging (LIDAR)	0.905 μm	Error when visibility < 4 m; Failure when visibility < 1 m
Ultrasound	~6.8 mm (50 kHz)	Limited influence after calibration
Radar	~11.5 mm (26 GHz)	Limited influence

**Table 2:** The main technical parameters of HC-SR04.

**Operating Voltage**	**Operating Current**	**Operating Frequency**	**Measurement Angle**
DC 5V	15 mA	40 kHz	15°
**Maximum Distance**	**Minimum Distance**	**Input Trigger Signal**	**Output Response Signal**
400 cm	2 cm	10 μs transistor-transistor logic (TTL) pulse	TTL level signal

**Table 3:** Statistical analysis of measurement errors for the three methods.

Method	Maximum Error (cm)	Minimum Error (cm)	Mean Absolute Deviation (cm)	Standard Deviation (cm)	Maximum Relative Errors
Constant speed	6.25	0.1	1.9	1.8	7.81%
Temperature compensation	8.3	0.3	3.6	2.0	10.4%
Baffle calibration	2.2	0.04	1.0	0.6	2.75%

Note: All the measurements were taken after 200 s after the smoke environment is stable.

**Table 4:** Statistical analysis of measurement errors for the three methods.

Method	Maximum Error (cm)	Minimum Error (cm)	Mean Absolute Deviation (cm)	Standard Deviation (cm)	Maximum Relative Errors
Constant speed	6.7	1.6	3.2	0.69	8.4%
Temperature compensation	4.1	0.8	1.6	0.61	5.12%
Baffle calibration	2.1	0.02	0.9	0.44	2.62%

Note: All the measurements were taken after 400 s after the smoke is stable.
